# Supplementary material for: Delineating the early dissemination mechanisms of acral melanoma by integrating single-cell and spatial transcriptomic analyses
Source: Nat Commun. 2023 Dec 8;14:8119. doi: 10.1038/s41467-023-43980-y (PMC10709603; doi:10.1038/s41467-023-43980-y)
Supplement: Supplementary file 1 — Supplementary Information [file 41467_2023_43980_MOESM1_ESM.pdf]

Supplementary information

Delineating the Early Dissemination Mechanisms of Acral Melanoma by Integrating Single-Cell and Spatial Transcriptomic Analyses

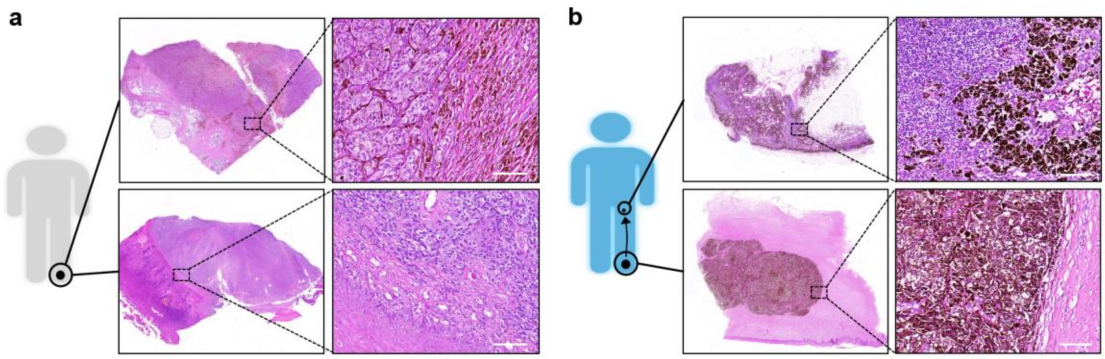

Supplementary Figure 1: Representative H&E images of enrolled patients in this study. a Representative H&E images of these primary AM tissues. Scale bar, 100 μm. **b** Representative H&E images of paired primary and metastatic LN tumor tissues. Scale bar, 100 μm. A part of the image has been adapted from Biorender.com.

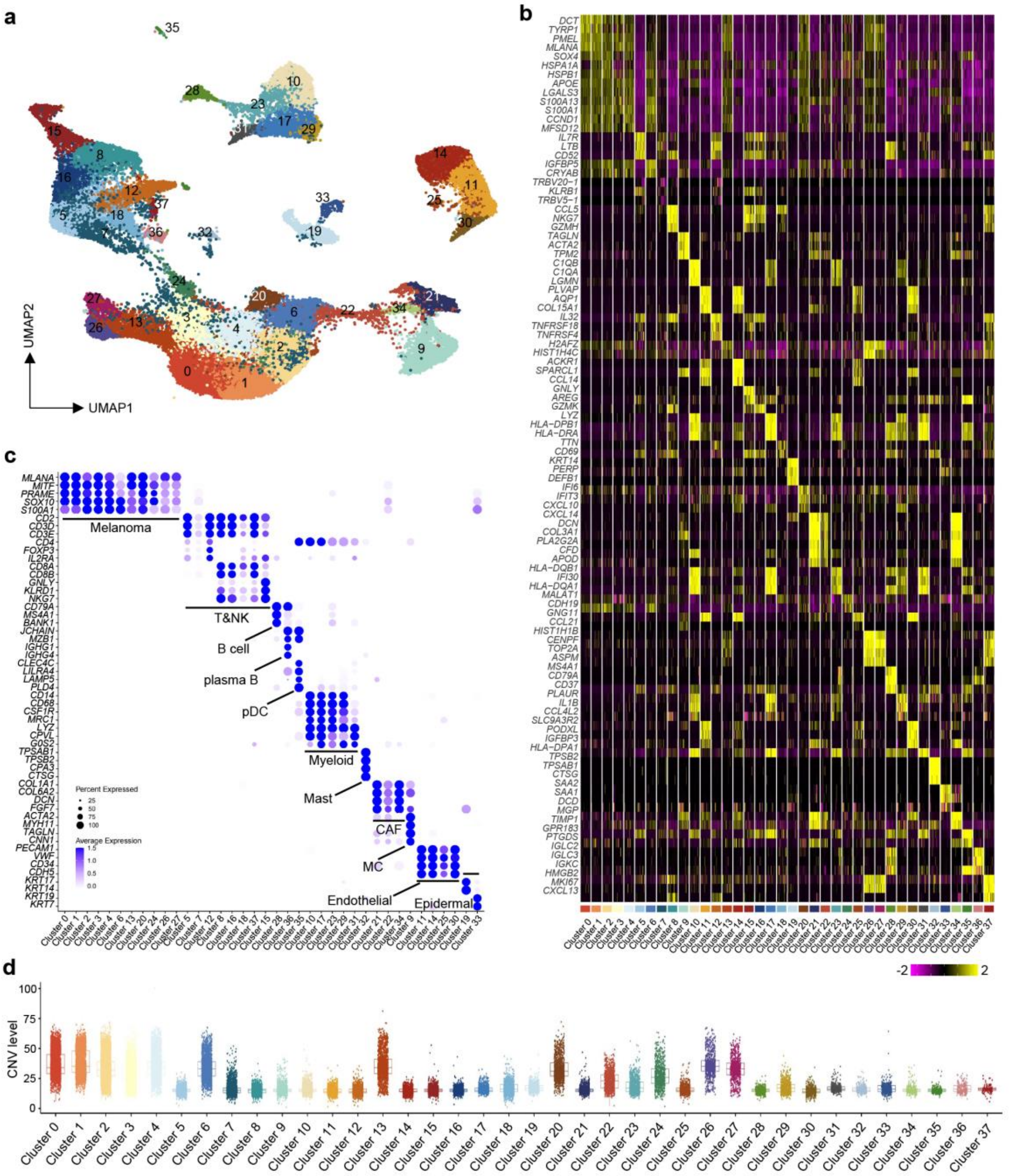

Supplementary Figure 2: The annotation of the AM ecosystem. a UMAP plot illustrating the annotated 38 clusters in the AM ecosystem. **b-c** Heatmap (b) and dot plot (c) displaying the cluster-specific marker genes used for annotation. Source data are provided in the Source Data file. **d** Box plot showing the median (middle line), 25th and 75th percentiles (box) and individual data points (single points) of CNV levels for the 38 annotated clusters.

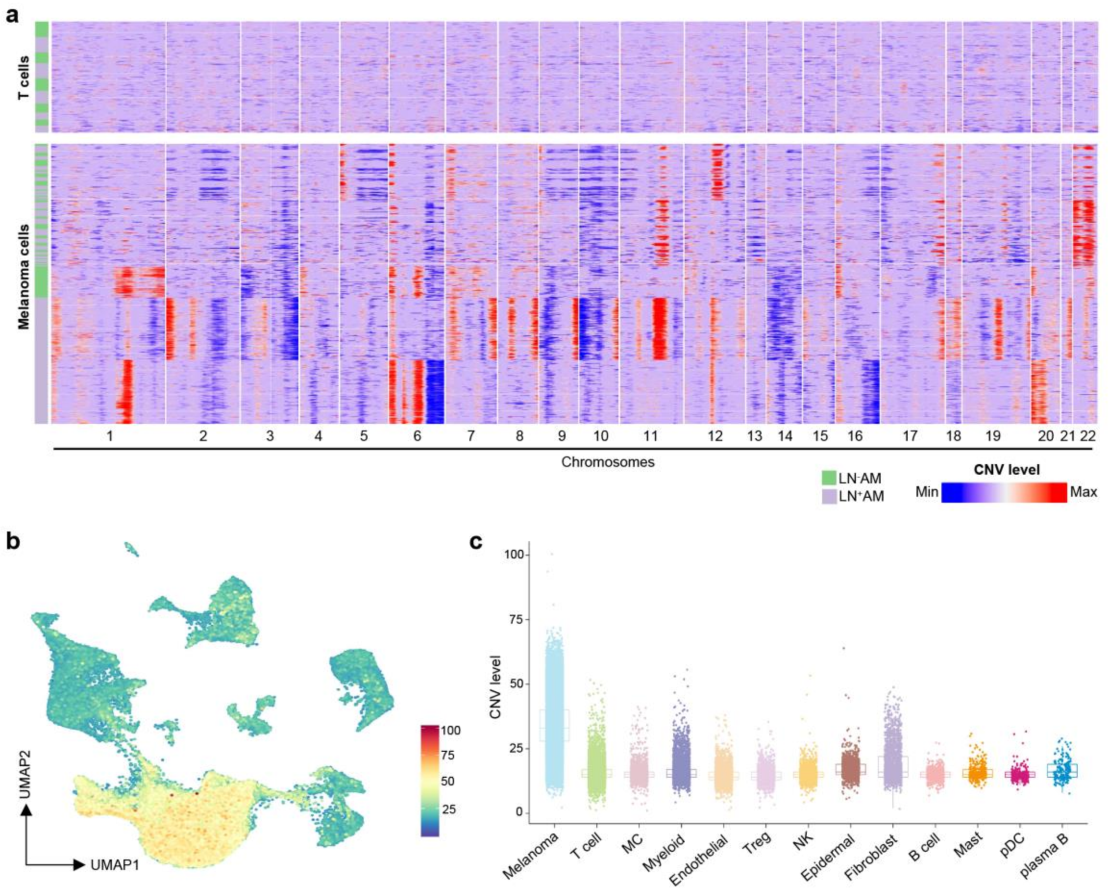

Supplementary Figure 3: Inference of malignant cells based on CNV levels. a Heatmap showing the distinct CNV levels of tumor cells and T cells (used as control) using the InferCNV analysis. **b** Feature plots displaying the CNV levels of these cells. **c** Box plot showing the median (middle line), 25th and 75th percentiles (box) and individual data points (single points) of CNV levels for the 13 cell types. Source data are provided in the Source Data file.

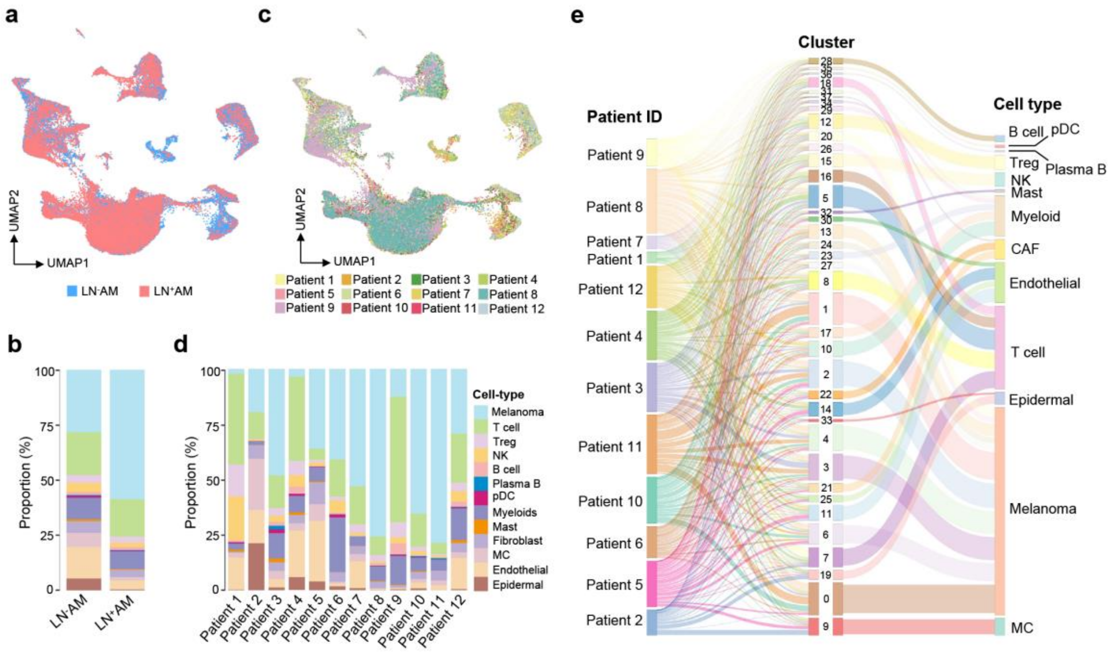

Supplementary Figure 4: High inter-tumoral heterogeneity of AM. a UMAP plot illustrating all cells in the tumor ecosystem, colored by $LN^{+}AM$ and $LN^{-}AM$. **b** Bar plot showing the fraction of cells in $LN^{+}AM$ and $LN^{-}AM$. Source data are provided in the Source Data file. **c** UMAP plot depicting all cells in the tumor ecosystem, colored by patients. **d** Bar plot showing the fraction of cells in each patient. Source data are provided in the Source Data file. **e** Sankey plot displaying the congruent relationship between patients, cell clusters, and cell types.

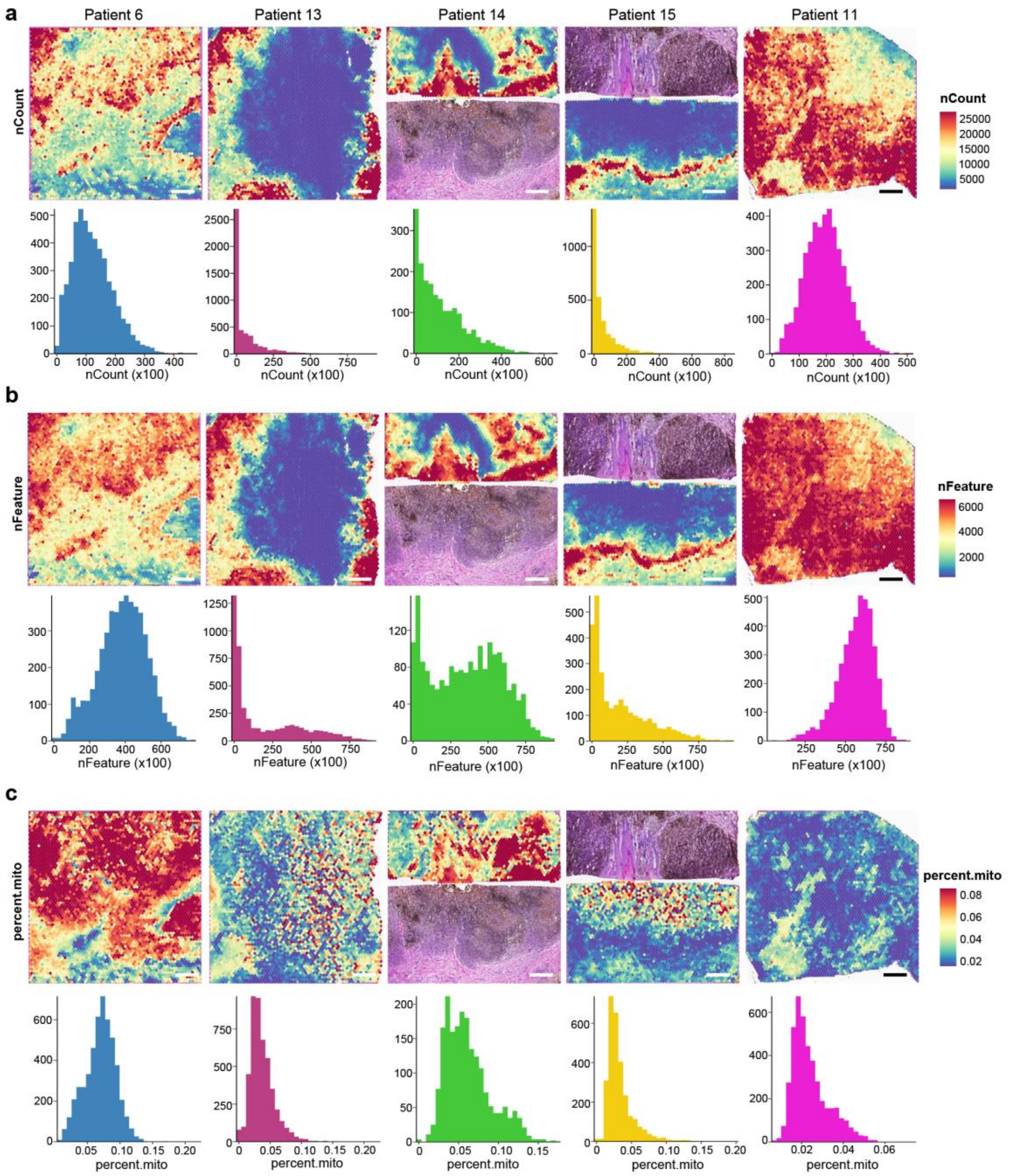

Supplementary Figure 5: High quality spots were enrolled in ST-seq data. a-c Spatial feature plot illustrating the nCount (a), nFeature (b), and mitochondrial gene proportion (c) of tumor sections from the five AM patients. Scale bar, 1000 µm.

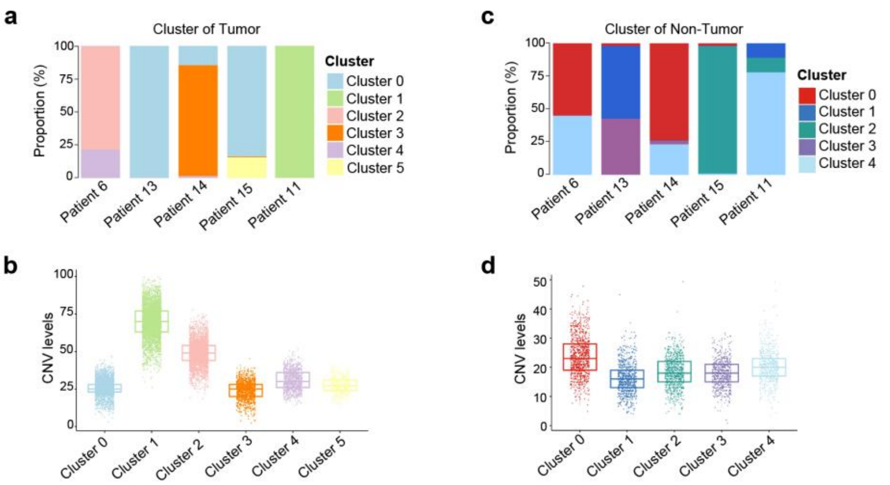

Supplementary Figure 6: Analysis of tumor and non-tumor regions in ST-seq data. a Bar plot showing the proportion of the six clusters of tumor regions among patients. Source data are provided in the Source Data file. **b** Box plot displaying the median (middle line), 25th and 75th percentiles (box), and individual data points (single points) of CNV levels in the six clusters of tumor regions. Source data are provided in the Source Data file. **c** Bar plot illustrating the proportion of the five clusters of non-tumor regions among patients. Source data are provided in the Source Data file. **d** Box plot showing the median (middle line), 25th and 75th percentiles (box), and individual data points (single points) of CNV levels in the five clusters of non-tumor regions. Source data are provided in the Source Data file.

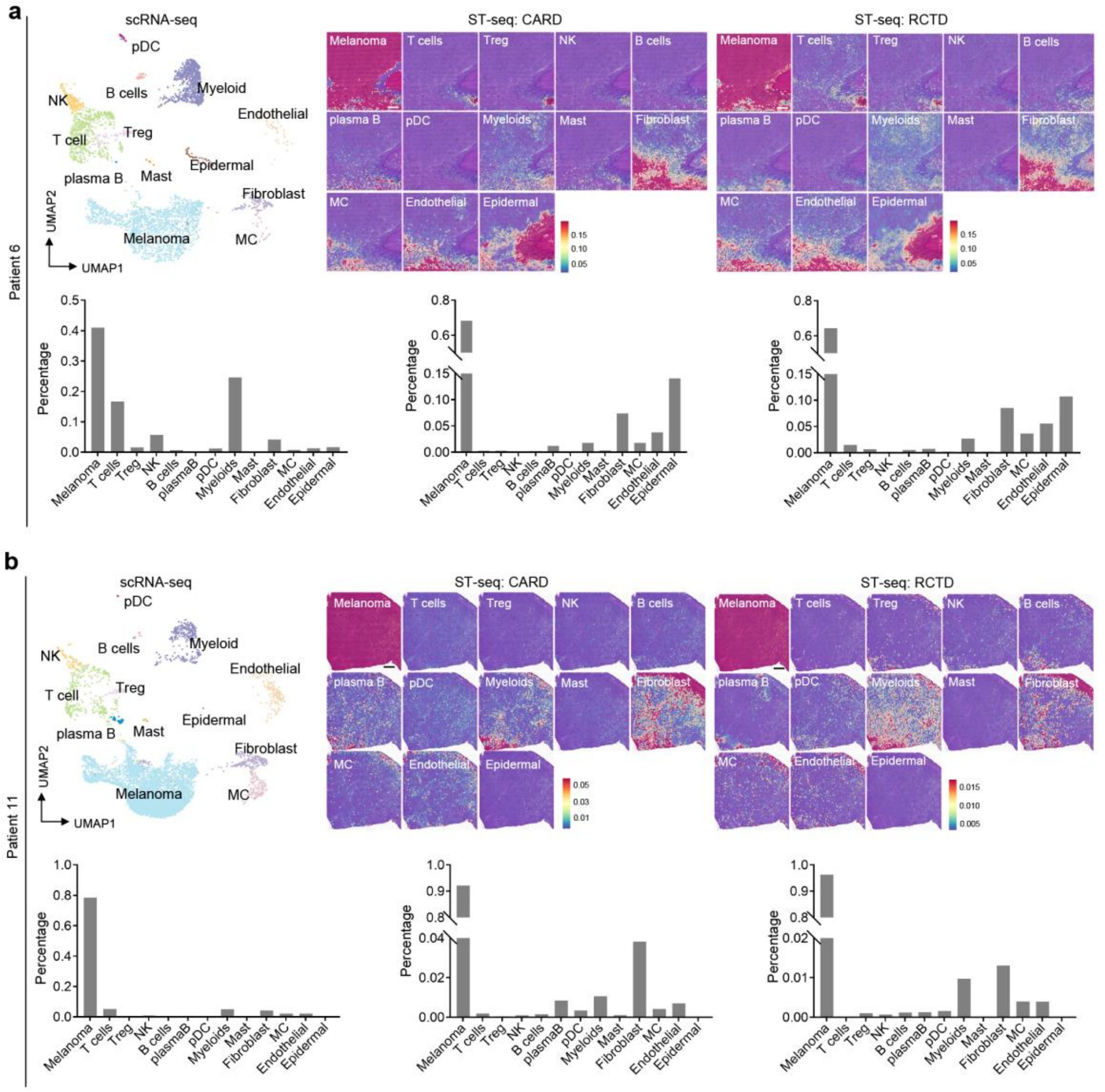

Supplementary Figure 7: The paired scRNA-seq and ST-seq data of patient 6 and 11. a, b UMAP plot and spatial feature plot (using CARD and RCTD methods) illustrating the distribution of the annotated 13 cell types, and histogram showing the percentage of these cells in patient 6 (a) and patient 11 (b). Scale bar, 1000 μm. Source data are provided in the Source Data file.

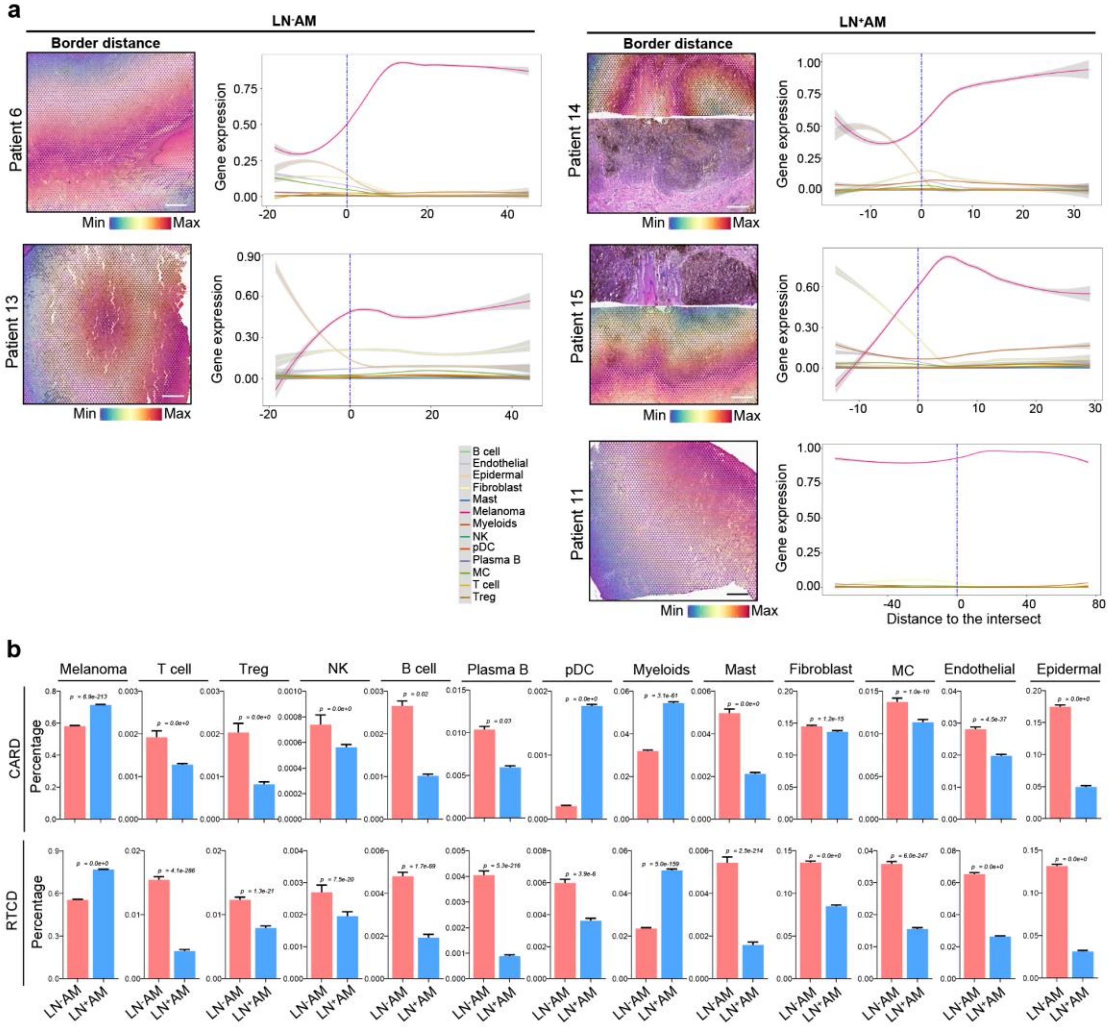

Supplementary Figure 8: Spatial distribution of the annotated cell types. a Cell percentage along the spatial distribution in each tissue section, as determined by the RCTD method. The error bands represent the 95% confidence interval. Source data are provided in the Source Data file. Scale bar, 1000 μm. **b** Histogram showing the proportion of cells in the LN^{+}AM and LN^{-}AM groups based on ST-seq data. The error bars represent the means ± SEM. Significance was determined using a two-sided Mann-Whitney U-test. Source data are provided in the Source Data file.

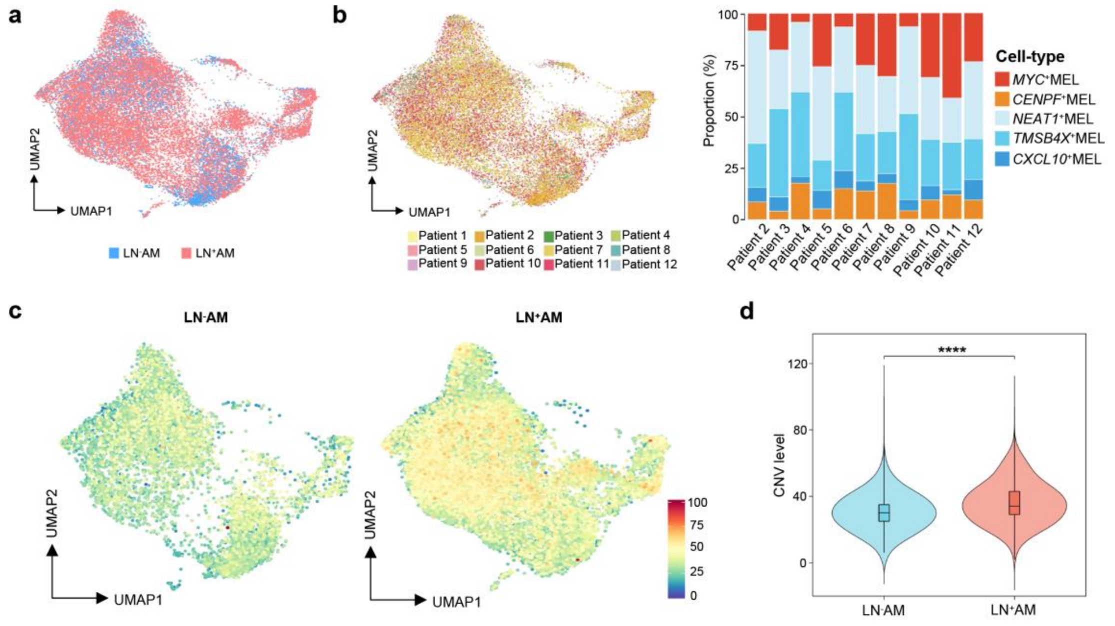

Supplementary Figure 9: Single-cell and spatial transcriptomics of melanoma subclusters. a UMAP plot illustrating the distribution of melanoma cells, colored by $LN^{-}AM$ and $LN^{+}AM$. **b** UMAP plot displaying the distribution of melanoma cells, colored by patients. The bar plot shows the proportion of melanoma subclusters among different patients. Source data are provided in the Source Data file. **c** Feature plot showing the CNV levels of tumor cells, split by $LN^{+}AM$ and $LN^{-}AM$. **d** Box plot showing the CNV levels of tumor cells in LN^{-} and LN^{+} AM. The center lines, bounds of the box, and whiskers indicate the medians, first and third quartiles, and minimum and maximum values within 1.5 x IQR of the box limits, respectively. Significance was determined using a two-sided Mann-Whitney U-test (**** *P*-value < 0.0001, *P* = *7.3e-295*). Source data are provided in the Source Data file.

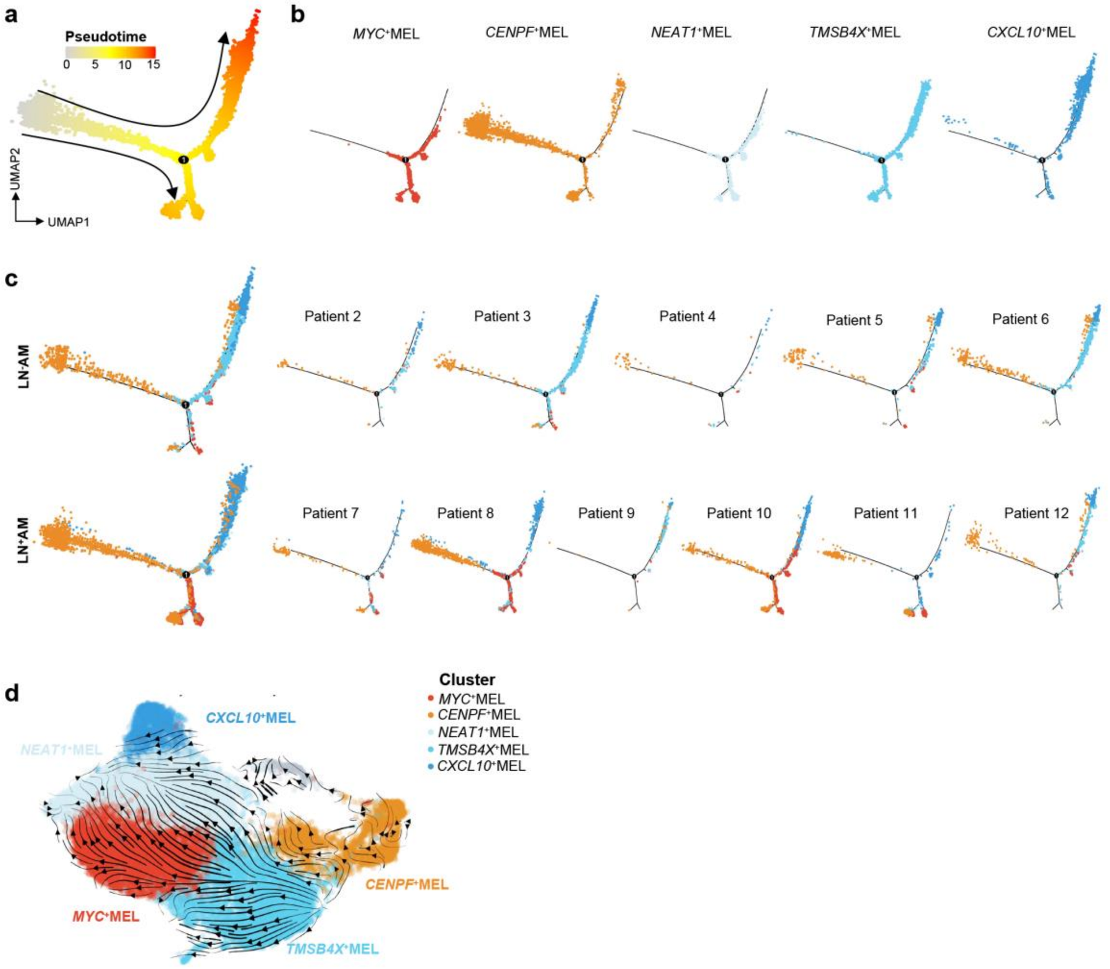

Supplementary Figure 10: Evolutionary trajectories of distinct AM subclusters. a Trajectory plot illustrating the evolutionary trajectory of all tumor cells, ordered by pseudotime. **b** Trajectory plot showing the evolutionary trajectory of all tumor cells, split by the five melanoma subclusters. **c** Trajectory plot displaying the evolutionary trajectory of all tumor cells, split by LN^{-}AM and LN^{+}AM, as well as by each patient. **d** RNA velocity showing the evolutionary trajectory of all tumor cells, colored by the melanoma subclusters.

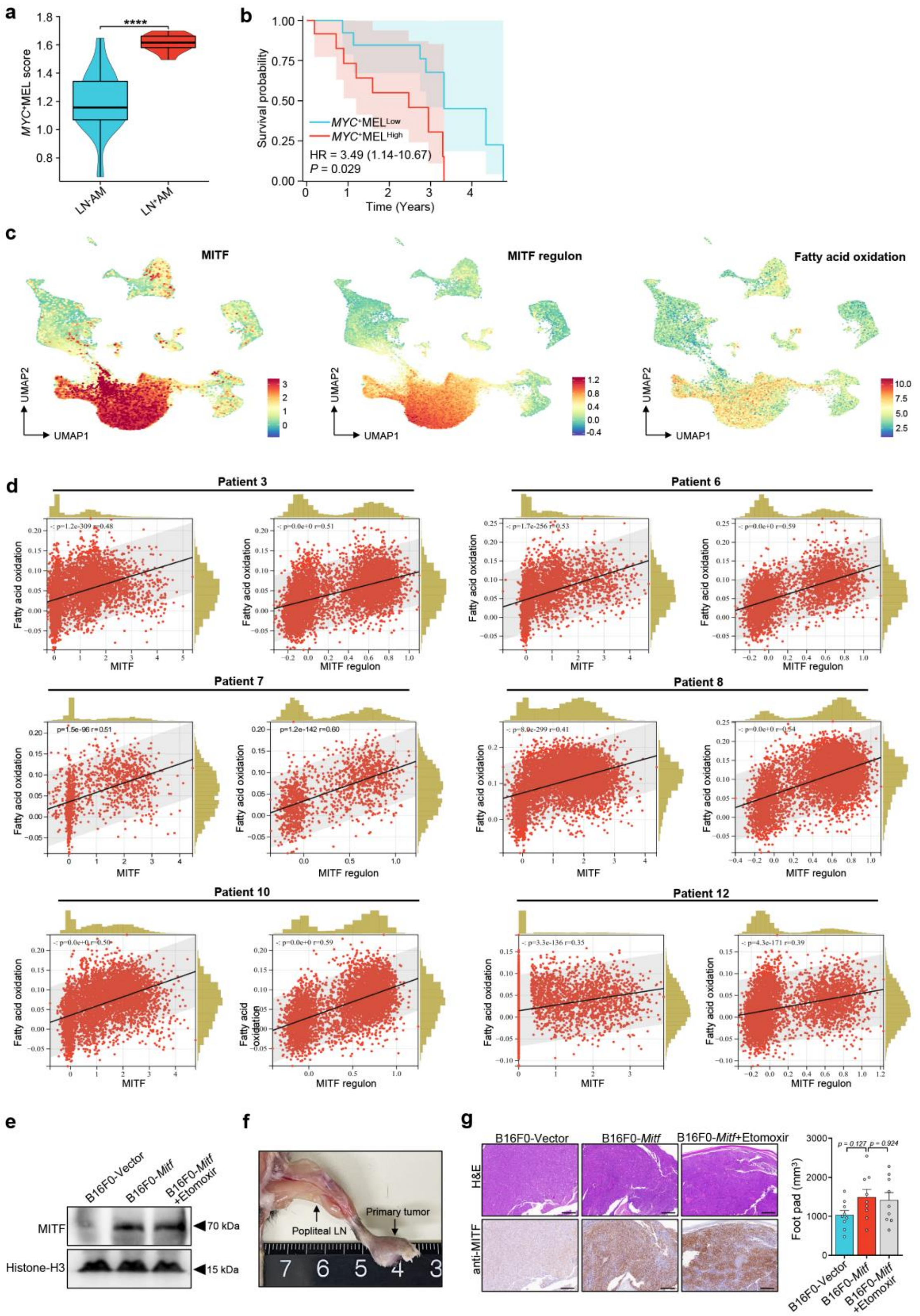

Supplementary Figure 11: Positive correlation between MITF and FAO. a Box plot showing the median (middle line), 25th, 75th percentiles (box) of *MYC*$^{+}$MEL score in LN^{-}AM (n = 16) and LN^{+}AM (n = 10). Significance was determined using an unpaired two-sided student's t-test (**** *P*-value < 0.0001, *P* = 6.8e-7). Source data

are provided in the Source Data file. **b** Kaplan-Meier analysis showing the overall survival rate of 26 AM patients with high and low levels of *MYC*$^{+}$MEL score using the log-rank test (*P*-value = 0.029, HR = 3.49 (1.14-10.67)). Source data are provided in the Source Data file. **c** Feature plots showing the expression of *MITF*, the activity of MITF regulon, and FAO score in AM. **d** Scatter plot showing the correlation between *MITF* and MITF regulon activity (x-axis) with FAO score (y-axis) in selected patients. The correlation is evaluated using the Spearman correlation coefficient. Source data are provided in the Source Data file. **e** Immunoblotting was performed to detect the levels of MITF and Histone-H3 in the indicated cells, and representative bands are shown. The experiment was repeated three times with similar results. **f** Representative images of primary tumor and metastatic popliteal LN. **g** H&E and anti-MITF stained images of the primary tumor tissues in the indicated groups (n = 9). The sizes of footpad were calculated using the histogram. Significance was determined using a one-way ANOVA test. Error bars represent the means ± SD. Scale bar, 500 µm. Source data are provided in the Source Data file.

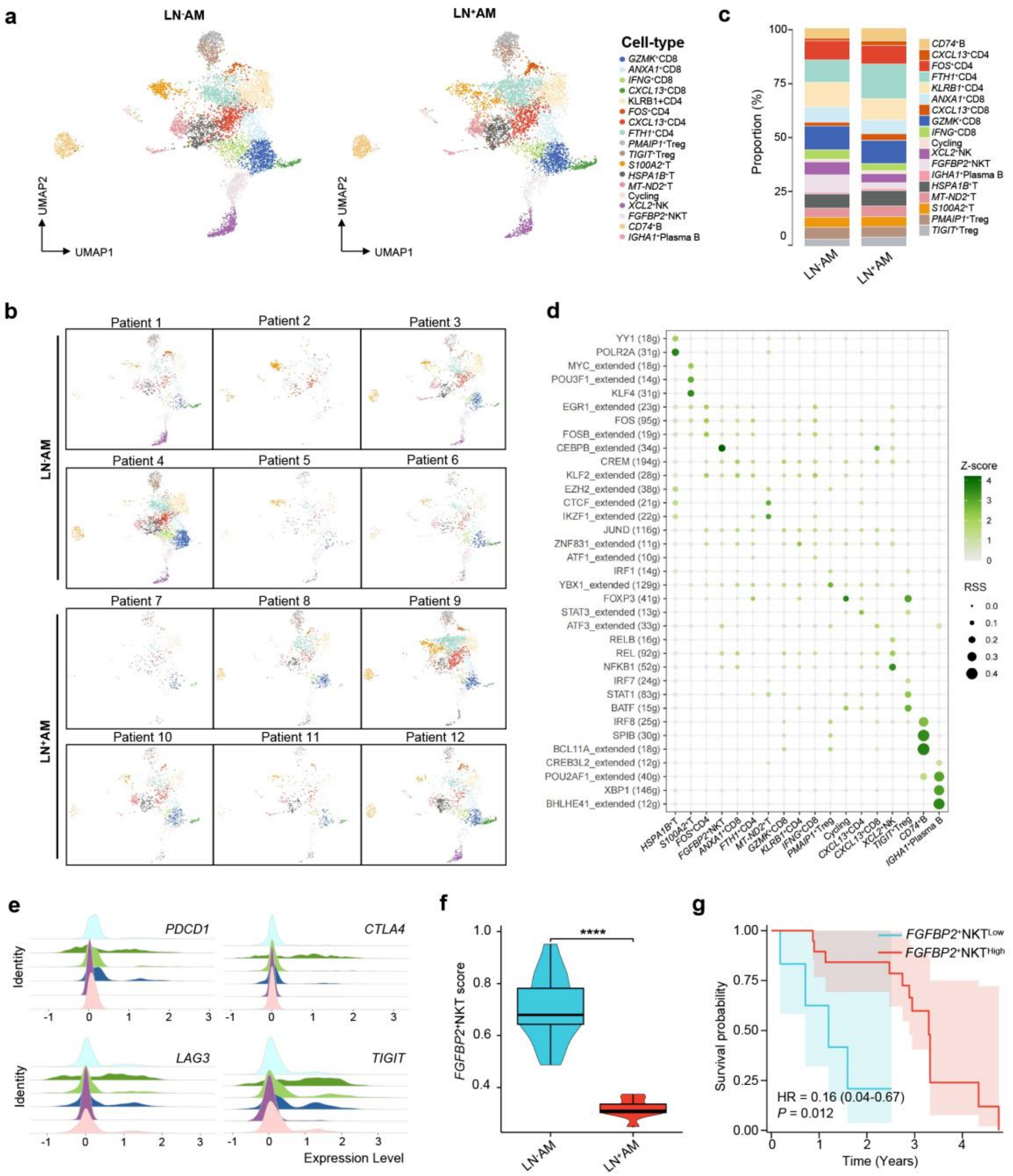

Supplementary Figure 12: Single-cell profiles of immune cells. a UMAP plot displaying the distribution of immune cells in LN$^-$AM and LN$^+$AM, colored by the annotated subclusters. **b** UMAP plot illustrating the distribution of immune cells in each patient, colored by the annotated subclusters. **c** Bar plot showing the fraction of annotated cell types in LN$^+$AM and LN$^-$AM. Source data are provided in the Source Data file. **d** Bubble plot showing the expression of key transcription factors (estimated using SCENIC) among the subclusters of immune cells. **e** Ridge plots showing the expression of *PDCD1*, *CTLA4*, *LAG3*, and *TIGIT* in the immune cell subclusters. **f**

Box plot showing the median (middle line), 25th, 75th percentiles (box) of *FGFBP2*$^{+}$NKT score in LN^{-}AM (n = 16) and LN^{+}AM (n = 10). Significance was determined using an unpaired two-sided student's t-test (**** *P*-value < 0.0001, *P* = 7.1e-10). Source data are provided in the Source Data file. **g** Kaplan-Meier analysis showing the overall survival rate of 26 AM patients with high and low levels of *FGFBP2*$^{+}$NKT score using the log-rank test (*P*-value = 0.012, HR = 0.16 (0.04-0.67)). Source data are provided in the Source Data file.

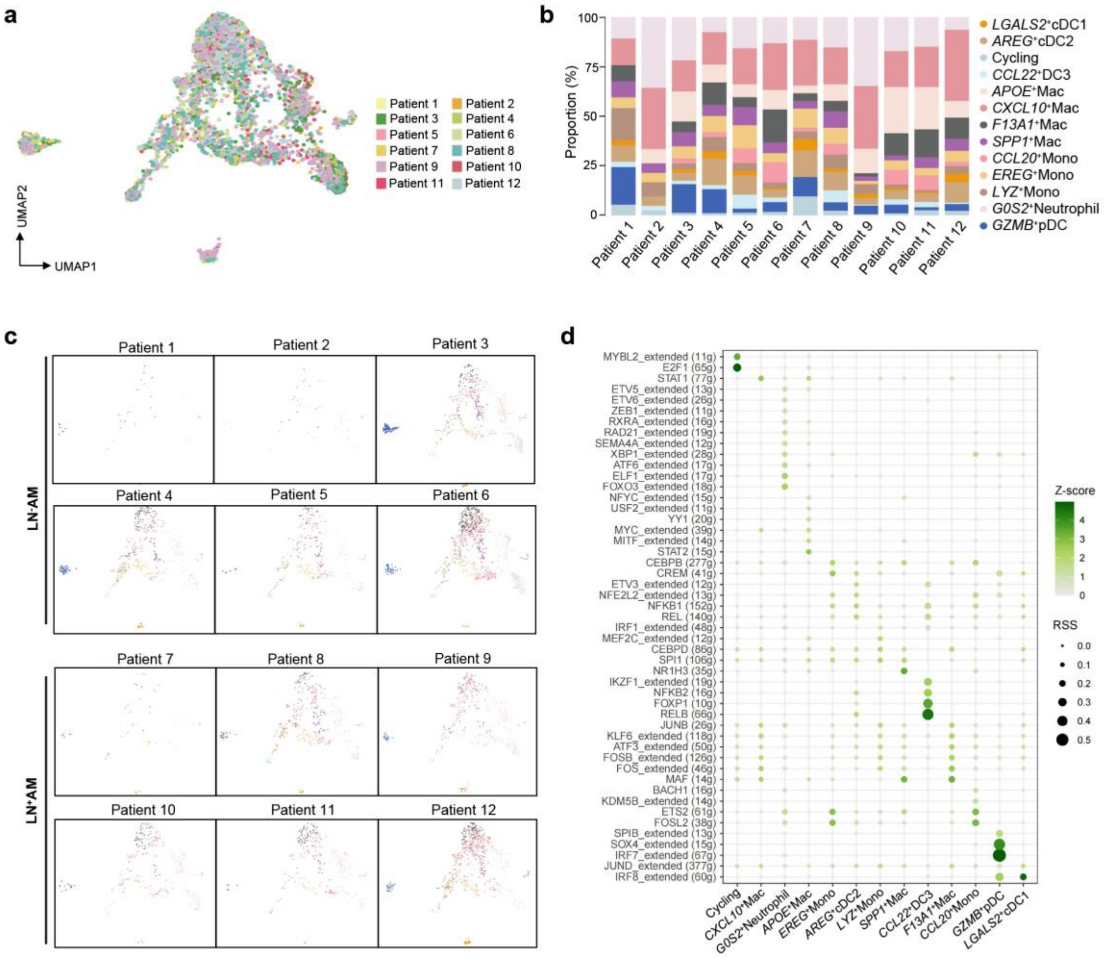

Supplementary Figure 13: Single-cell profiles of myeloid cells. a UMAP plot illustrating all myeloid cells, colored by patients. **b** Bar plot showing the proportion of myeloid subclusters in each patient. Source data are provided in the Source Data file. **c** UMAP plot displaying myeloid cells in each patient, colored by the annotated subclusters. **d** Bubble plot showing the expression of key transcription factors (estimated using SCENIC) of myeloid subclusters.

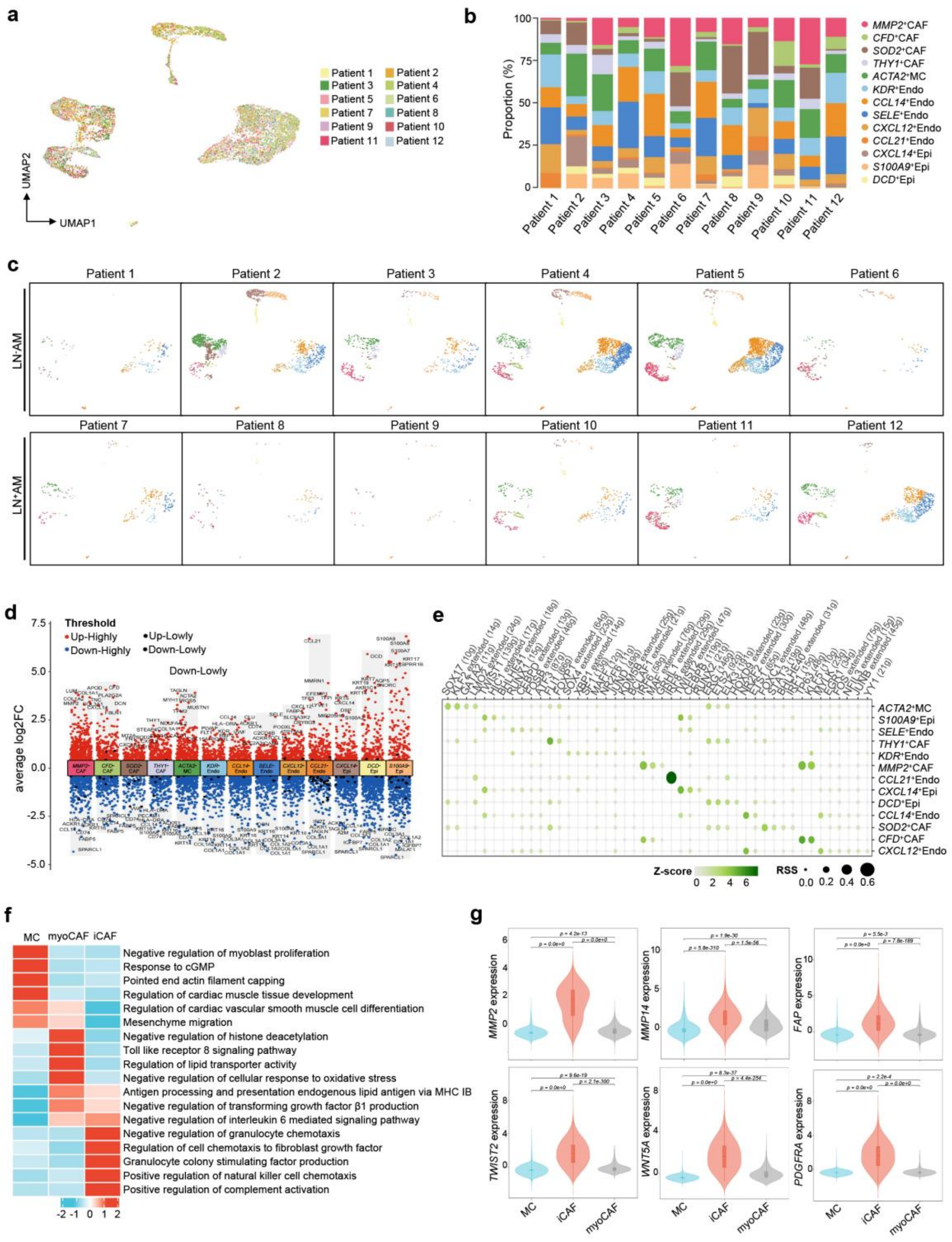

Supplementary Figure 14: Single-cell profiles of stromal cells. a UMAP plot depicting all stromal cells, colored by patients. **b** Bar plot showing the proportion of subclusters of stromal cells in each patient. Source data are provided in the Source Data file. **c** UMAP plot illustrating all stromal cells in each patient, colored by the annotated subclusters. **d** Volcano plot displaying the differentially expressed genes in the annotated subclusters. Source data are provided in the Source Data file. **e** Bubble

plot showing the expression of key transcription factors (estimated using SCENIC) between the subclusters of the stromal compartment. **f** Heatmap presenting the enriched pathways in MCs, myoCAFs and iCAFs. **g** Violin plot showing the expression levels of *MMP2*, *MMP14*, *FAP*, *TWIST2*, *WNT5A*, and *PDGFRA* in MCs, myoCAFs, and iCAFs. The box center lines, bounds of the box, and whiskers indicate medians, first and third quartiles, and minimum and maximum values within 1.5 x IQR of the box limits, respectively. Significance was determined using a one-way ANOVA test. (** *P*-value < 0.01, *** *P*-value < 0.001, **** *P*-value < 0.0001). Source data are provided in the Source Data file.

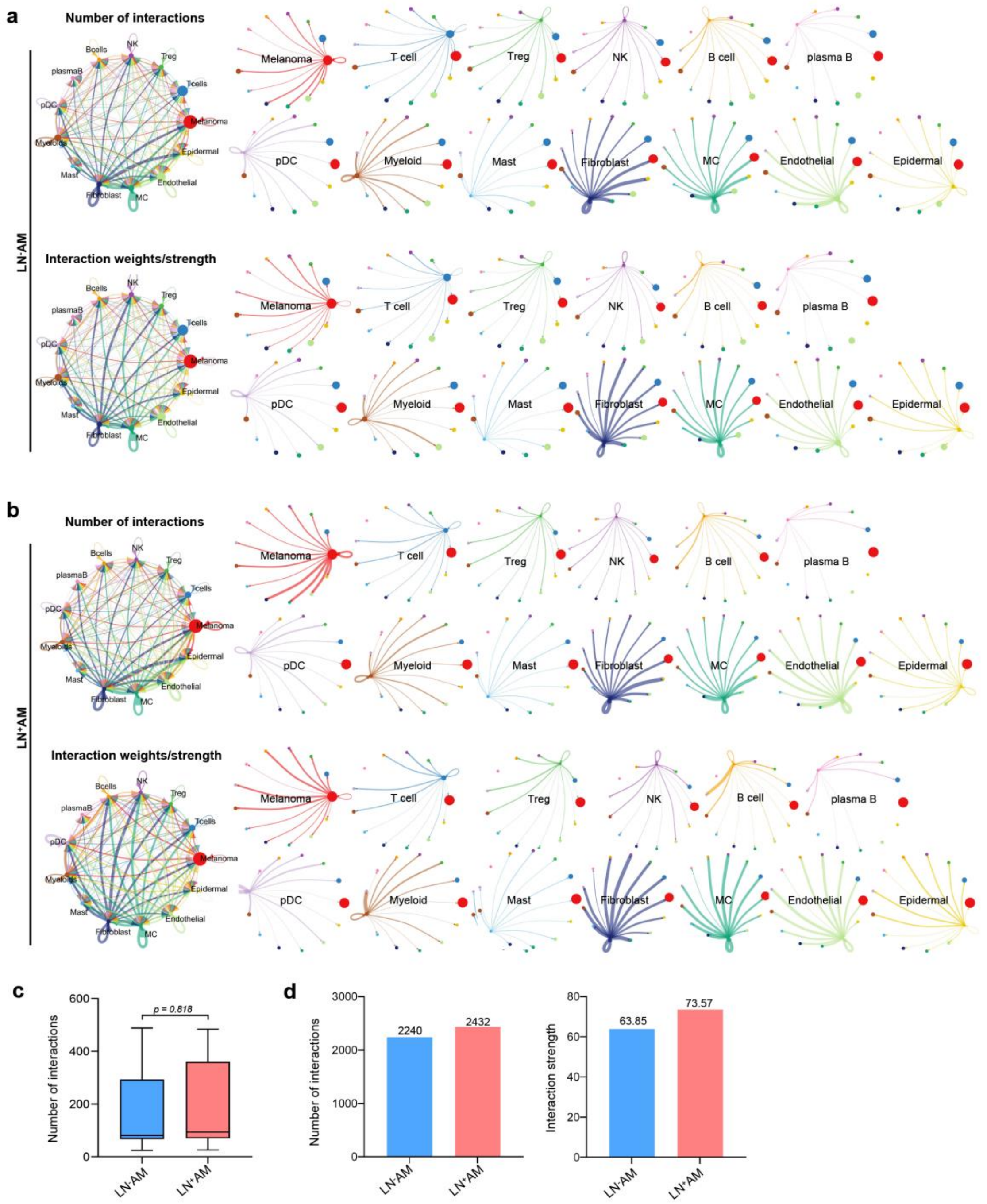

Supplementary Figure 15: Intercellular communication networks in AM. a, b The interaction number and weights/strength of the annotated 13 cell types are presented in LN$^-$AM (a) and LN$^+$AM (b). **c** Box plot showing the median (middle line), 25th and 75th percentiles (box) of the interaction number between the annotated 13 cell types in the scRNA-seq data. Significance was determined using an unpaired two-sided student's t-test. **d** Histogram displaying the interaction number and interaction strength of all cell types in LN$^-$AM and LN$^+$AM. Source data are provided in the Source Data file.

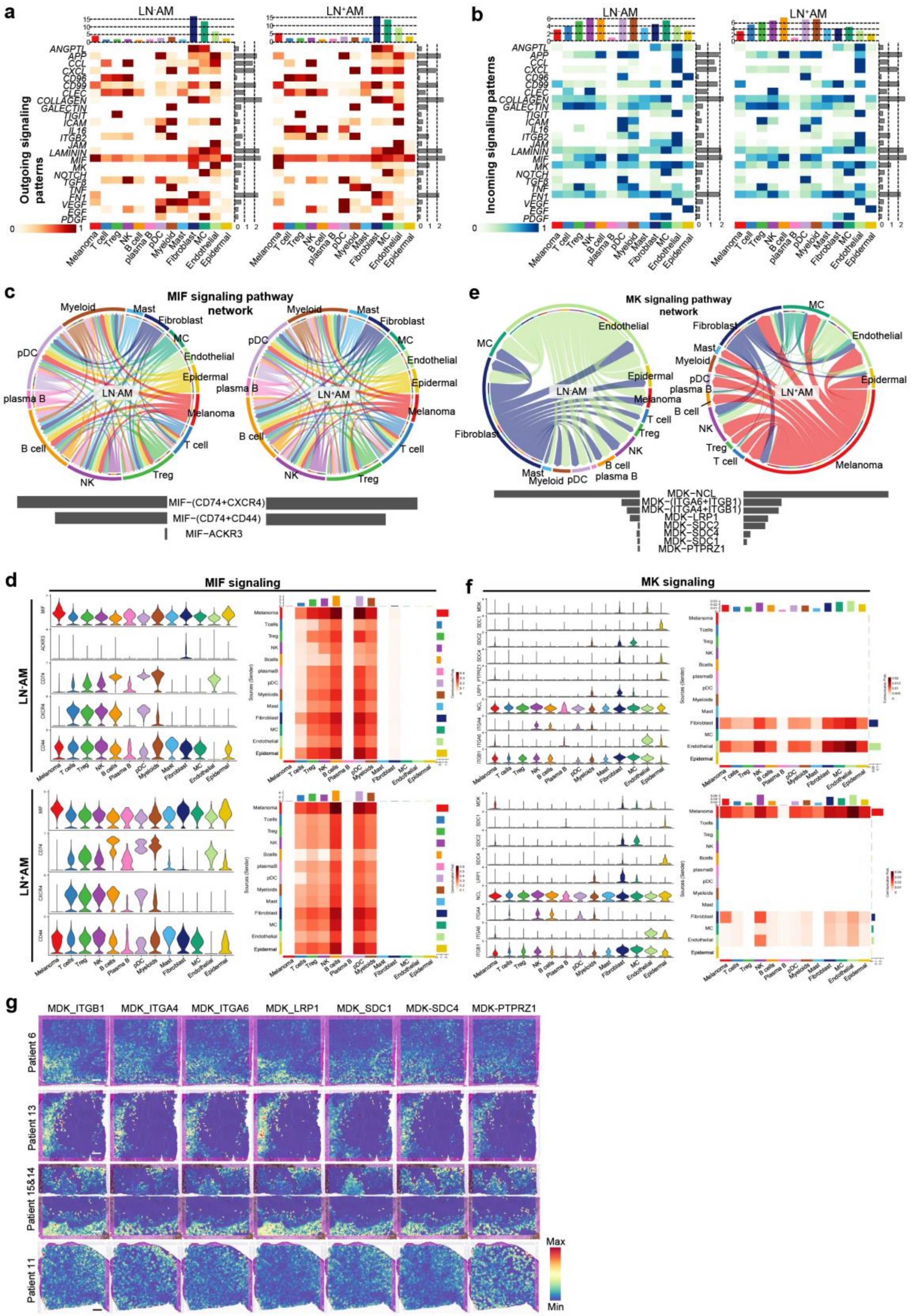

Supplementary Figure 16: Intercellular communication of selected signaling pathways. a, b Heatmap displaying the selected outgoing (a) and incoming (b) signaling patterns of all 13 cell types in LN$^-$AM and LN$^+$AM. Source data are provided in the Source Data file. **c** Chord plots presenting the MIF signaling networks

in LN^+AM and LN^-AM. Source data are provided in the Source Data file. **d** Violin plot showing the gene expression levels of L-R pairs in the MIF pathway, and the corresponding heatmap indicating the inferred communication probability among major cell types in LN^-AM and LN^+AM. Source data are provided in the Source Data file. **e** Chord plots illustrating the MK signaling networks in LN^+AM and LN^-AM. Source data are provided in the Source Data file. **f** Violin plot showing gene expression of L-R pairs in the MK pathway, and the corresponding heatmap indicating the inferred communication probability among major cell types in LN^-AM and LN^+AM. Source data are provided in the Source Data file. **g** Spatial feature plots showing the interaction activity of selected L-R pairs in the MK pathway using ST-seq data. Scale bar, 1000 μm.

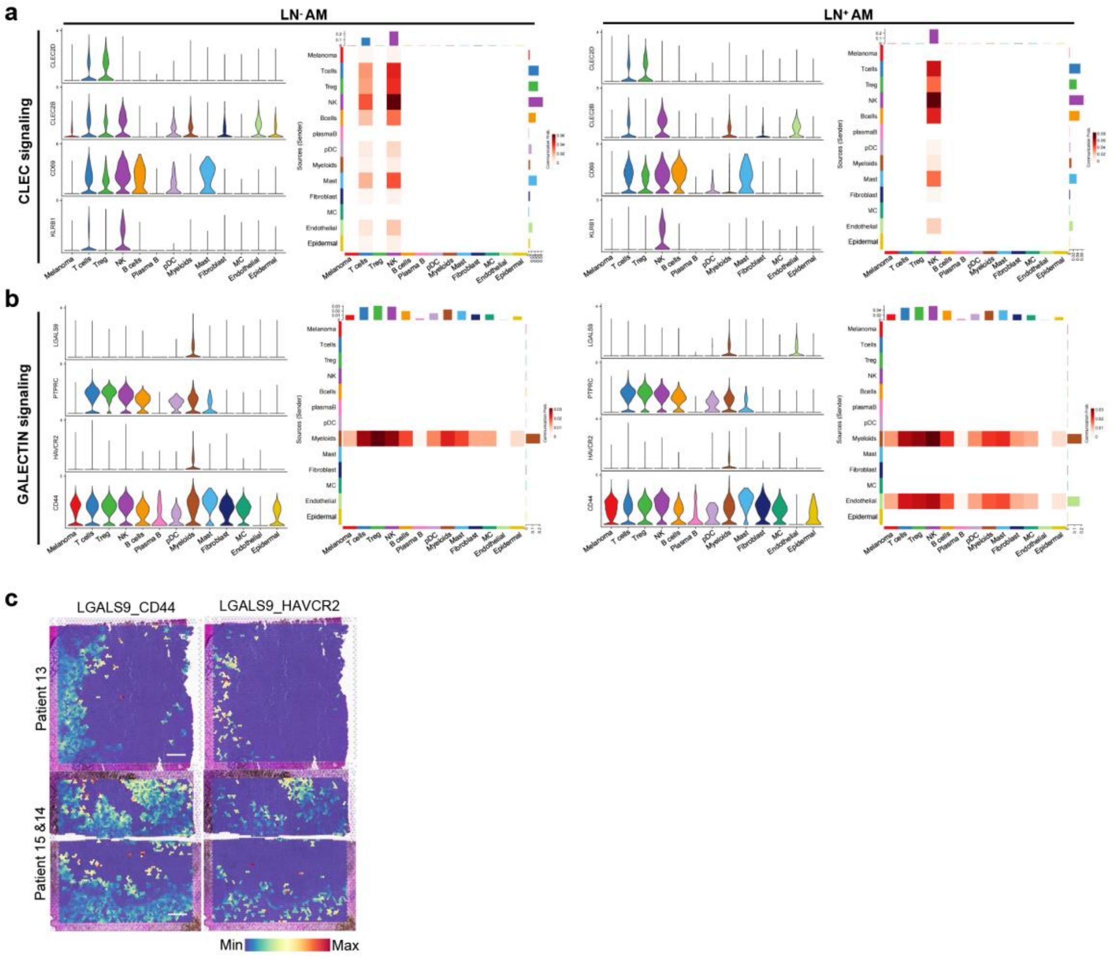

Supplementary Figure 17: Spatial feature plot of selected L-R pairs. a, b Violin plot depicting the gene expression levels of L-R pairs in the CLEC (a) and GALECTIN (b) pathways, and the accompanying heatmap indicating the inferred communication probability among major cell types in LN^{-}AM and LN^{+}AM. **c** Spatial feature plot showcasing the interaction activity of L-R pairs in the GALECTIN pathway using ST-seq data in five AM patients, and representative pictures were showed. Scale bar, 1000 μm.

Supplementary Table 1. Clinicopathological characteristics of AM patients in the scRNA-seq.

Sample ID	Age / Gender	Paimary Site	T-stage	N-stage	M-stage	TNM-stage	Preoperative treatment	Postoperative Treatment	Mutations identified	Sample source
Patient1	82/M	Left heel	T3b	N0	M0	IIB	No	No	no clinically mutations detected	GSE189889
Patien2	85/F	Left acrotarsium	T4b	N0	M0	IIC	No	No	KIT K642E	FDZSH
Patient3	57/M	Right acrotarsium	T4b	N0	M0	IIC	No	No	no clinically mutations detected	FDSCC
Patient4	64/M	Right pelma	T1a	N0	M0	IA	No	No	no clinically mutations detected	FDSCC
Patient5	46/F	Left heel	T4b	N0	M0	IIC	No	No	no clinically mutations detected	FDSCC
Patient6	69/M	Right heel	T4b	N0	M0	IIC	No	No	no clinically mutations detected	FDZSH
Patient7	70/F	Left first toe nail bed	T3b	N2a	M0	IIIC	No	Nivolumab	no clinically mutations detected	GSE189889
Patient8	90/F	Left first toe	T3b	N3a	M0	IIIC	No	No	no clinically mutations detected. VUS BRAF L711V	GSE189889
Patient9	68/M	Left first toe	T4b	N1b	M0	IIIC	No	Adjuvant Nivolumab	NF1 R1207* VAF: 17.7%	GSE189889
Patient10	63/M	Right 4th and 5th toe	T3a	N2b	M0	IIIB	No	No	no clinically mutations detected	FDSCC
Patient11	62/M	Right thumb	T4b	N2b	M0	IIIC	No	No	no clinically mutations detected	FDSCC
Patient12	62/M	Left palm	T4a	N3b	M0	IIIC	No	No	no clinically mutations detected	FDSCC

Supplementary Table 2. Clinicopathological characteristics of AM patients in the ST-seq.

Sample ID	Age / Gender	Paimary Site	T-stage	N-stage	M-stage	TNM-stage	Preoperative treatment	Postoperative Treatment	Mutations identified	Sample source
Patient6	69/M	Right heel	T4b	N0	M0	IIC	No	No	no clinically mutations detected	FDZSH
Patient11	62/M	Right thumb	T4b	N2b	M0	IIIC	No	No	no clinically mutations detected	FDSCC
Patient13	39/F	Right acrotarsium	T2a	N0	M0	IB	No	No	no clinically mutations detected	FDSCC
Patient14	53/M	Left acrotarsium	T4a	N3b	M0	IIIC	No	No	no clinically mutations detected	FDSCC
Patient15	72/M	Left acrotarsium	T4b	N3b	M0	IIID	No	No	no clinically mutations detected	FDSCC

\

Supplementary Table 3. Antibodies used in this study.

Antibody	Applications	Company	Cat#
MITF	IHC-P, WB, IP, ICC/IF, F	ABCAM	ab303530
CD4	IHC-P	Biolynx	BX50023
CD8	IHC-P	Biolynx	BX50036-C3
NCAM1 (CD56)	IHC-P, WB, IF, F	CST	3576S
FGFBP2	IHC-P, WB	SIGMA	HPA039180
FAP	IHC-P	ABCAM	ab207178
CD31	IHC-P	ABCAM	ab28364
Galectin-9	IHC-P, WB	NOVUS	NBP2-45619
TIM3	IHC-P, WB, IP, F	CST	45208S
CD45	IHC-P	Biolynx	BX50023
CD44	IHC-P, WB, IP, IF, F	CST	3570S
Histone-H3	IHC-P, WB, IP, IF, F	Proteintech	17168-1-AP

Abbreviations: WB, western blot; IHC-P, immunohistochemistry (Paraffin); ICC/IF, immunocytochemistry/immunofluorescence; IP, immunoprecipitation; F, Flow cytometric analysis;
