# Supplementary material for: Delineating the early dissemination mechanisms of acral melanoma by integrating single-cell and spatial transcriptomic analyses
Source: Nat Commun. 2023 Dec 8;14:8119. doi: 10.1038/s41467-023-43980-y (PMC10709603; doi:10.1038/s41467-023-43980-y)
Supplement: Supplementary file 3 — Reporting Summary [file 41467_2023_43980_MOESM3_ESM.pdf]

Corresponding author(s): Jianying Gu
Last updated by author(s): Nov 22, 2023

Reporting Summary

Nature Portfolio wishes to improve the reproducibility of the work that we publish. This form provides structure for consistency and transparency in reporting. For further information on Nature Portfolio policies, see our Editorial Policies and the Editorial Policy Checklist.

Statistics

For all statistical analyses, confirm that the following items are present in the figure legend, table legend, main text, or Methods section.

n/a	Confirmed	
☐	☒	The exact sample size (*n*) for each experimental group/condition, given as a discrete number and unit of measurement
☐	☒	A statement on whether measurements were taken from distinct samples or whether the same sample was measured repeatedly
☐	☒	The statistical test(s) used AND whether they are one- or two-sided<br>*Only common tests should be described solely by name; describe more complex techniques in the Methods section.*
☐	☒	A description of all covariates tested
☐	☒	A description of any assumptions or corrections, such as tests of normality and adjustment for multiple comparisons
☐	☒	A full description of the statistical parameters including central tendency (e.g. means) or other basic estimates (e.g. regression coefficient) AND variation (e.g. standard deviation) or associated estimates of uncertainty (e.g. confidence intervals)
☐	☒	For null hypothesis testing, the test statistic (e.g. F, t, r) with confidence intervals, effect sizes, degrees of freedom and P value noted<br>*Give P values as exact values whenever suitable.*
☒	☐	For Bayesian analysis, information on the choice of priors and Markov chain Monte Carlo settings
☒	☐	For hierarchical and complex designs, identification of the appropriate level for tests and full reporting of outcomes
☐	☒	Estimates of effect sizes (e.g. Cohen's *d*, Pearson's *r*), indicating how they were calculated

Our web collection on statistics for biologists contains articles on many of the points above.

Software and code

Policy information about availability of computer code

Data collection	No specialized software was used for data acquisition.
Data analysis	All software used in this study are published and cited either in the main text or Online method. Here is the list of software used in this study. R (v4.1.1), GraphPad Prism (v9.0), Cell Ranger (v5.0.0), Seurat (v3.1.1), DoubletFinder (v2.0.2), inferCNV (v1.0.4), Monocle2 (v2.9.0), velocyto.R (v0.6), SCENIC (v1.1.2), CellChat (v1.1.3), GSVA (v1.30.0), GSEABase (v1.44.0), LIMMA (v3.38.3), Spaceranger (v1.2.0), ComplexHeatmap (v2.15.4), cowplot (v1.1.1), data.table (v1.14.8), dplyr (v1.1.2), future (v1.32.0), future.apply (v1.11.0), ggplot2 (v3.4.2), irGSEA (v2.1.5), magrittr (v2.0.3), Matrix (v1.5-4), msigdbr (v7.5.1), patchwork (v1.1.2), RColorBrewer (v1.1-3), SPOTlight (v1.5.1), STdeconvolve (v1.3.1), tidyr (v1.3.0), circlize (v0.4.15), clusterProfiler (v4.6.2), ConsensusClusterPlus (v1.62.0), cowplot (v1.1.1), datasets (v4.3.0), dbplyr (v2.3.2), DESeq2 (v1.38.3), devtools (v2.4.5), DOSE (v3.24.2), DoubletFinder (v2.0.3), edgeR (v3.40.2), enrichplot (v1.18.4), fgsea (v1.24.0), garnett (v0.1.23), ggExtra (v0.10.0), ggpubr (v0.6.0), ggsci (v3.0.0), graphics (v4.3.0), GSVA (v1.46.0), harmony (v0.1.1), org.Hs.eg.db (v3.16.0), pheatmap (v1.0.12), rlang (v1.1.1), spacexr (v2.2.0), survival (v3.5-5).

For manuscripts utilizing custom algorithms or software that are central to the research but not yet described in published literature, software must be made available to editors and reviewers. We strongly encourage code deposition in a community repository (e.g. GitHub). See the Nature Portfolio guidelines for submitting code & software for further information.

Data

Policy information about availability of data

All manuscripts must include a data availability statement. This statement should provide the following information, where applicable:

- Accession codes, unique identifiers, or web links for publicly available datasets
- A description of any restrictions on data availability
- For clinical datasets or third party data, please ensure that the statement adheres to our policy

The raw sequence data reported in this paper has been deposited in the Genome Sequence Archive (Genomics, Proteomics & Bioinformatics 2021) in National Genomics Data Center (Nucleic Acids Res 2022), China National Center for Bioinformation / Beijing Institute of Genomics, Chinese Academy of Sciences under the accession number HRA004456, which is publicly accessible (https://ngdc.cncb.ac.cn/gsa-human/browse/HRA004456). The raw sequencing data is available for non-commercial purposes under controlled access because of data privacy laws, and access can be obtained by request to the corresponding authors. For public datasets analysis, Li et al.'s dataset (including 4 AM samples) were retrieved from GSE189889 (https://www.ncbi.nlm.nih.gov/geo/query/acc.cgi?acc=GSE189889). The bulk RNA-seq data of 26 primary AM samples were retrieved from GSE162682 (https://www.ncbi.nlm.nih.gov/geo/query/acc.cgi?acc=GSE162682). Raw sequencing reads were mapped, annotated, and quantified using the GRCh38 reference annotation file (https://cf.10xgenomics.com/supp/cell-exp/refdata-gex-GRCh38-2020-A.tar.gz). The remaining data are available within the Article, Supplementary Information or Source Data file. Source data are provided with this paper.

Research involving human participants, their data, or biological material

Policy information about studies with human participants or human data. See also policy information about sex, gender (identity/presentation), and sexual orientation and race, ethnicity and racism.

Reporting on sex and gender	For scRNA-seq, 8 patients were male, and 4 patients were female. For ST-seq, 4 patients were male and one was female. Samples from two patients were used in both scRNA-seq and ST-seq. Detailed clinicopathologic characteristics of these patients were summarized in the Supplementary Data 1 and Supplementary Data 2.
Reporting on race, ethnicity, or other socially relevant groupings	For scRNA-seq, 8 patients' race from FDZSH and FDSCC were Yellow, and 4 patients' race from GSE189889 are white. For ST-seq, 5 patients' race from FDZSH and FDSCC are Yellow. Detailed clinicopathologic characteristics of these patients were summarized in the Supplementary Data 1 and 2.
Population characteristics	For scRNA-seq, 12 patients ranged in age from 46 to 90. For ST-seq, 5 patients ranged in age from 39 to 72. Detailed clinicopathologic characteristics of these patients were summarized in the Supplementary Data 1 and 2.
Recruitment	For scRNA-seq, a total of 12 AM patients were enrolled in this study. Eight patents who underwent curative surgical resection during October 2021 to January 2022 from the Department of Plastic & Reconstructive Surgery, Zhongshan Hospital of Fudan University (FDZSH) and the Department of Musculoskeletal Oncology, Fudan University Shanghai Cancer Center (FDSCC) were randomly selected. Four AM patents were downloaded from the GEO database under accession number GSE189889. Detailed clinicopathologic characteristics of these patients were summarized in the Supplementary Data 1. Five AM patients undergoing curative surgical resection were randomly enrolled from the Department of Plastic & Reconstructive Surgery of FDZSH and the Department of Musculoskeletal Oncology of FDSCC. Detailed clinicopathologic characteristics of these patients were summarized in the Supplementary Data 2. Tumor cells in primary and metastatic lesions were confirmed by pathologists through both cytological detections during the surgery and the paraffin section after surgery. None of these patients received targeted therapy, immunotherapy, or any other anti-tumor therapy before surgery.
Ethics oversight	This study was conducted in accordance with ethical standards of the Institutional Review Board of FDZSH and FDSCC (B2023-049R). Written informed consents were obtained from all patients involved in this study for the use of their tissue samples and clinical information.

Note that full information on the approval of the study protocol must also be provided in the manuscript.

Field-specific reporting

Please select the one below that is the best fit for your research. If you are not sure, read the appropriate sections before making your selection.

☒ Life sciences ☐ Behavioural & social sciences ☐ Ecological, evolutionary & environmental sciences

For a reference copy of the document with all sections, see nature.com/documents/nr-reporting-summary-flat.pdf

Life sciences study design

All studies must disclose on these points even when the disclosure is negative.

Sample size	For scRNA-seq, a total of 12 AM patients undergoing curative surgical resection were randomly enrolled in this study, including 8 from the FDZSH and the FDSCC, and 4 were downloaded from the GEO database under accession number GSE189889. Since the incidence rate of AM is low, 6 cases with LN metastasis and 6 cases without LN metastasis were selected to ensure a comprehensive statistical analysis. For ST-seq, 5 AM patients undergoing curative surgical resection were randomly enrolled from the Department of Plastic & Reconstructive Surgery of FDZSH and the Department of Musculoskeletal Oncology of FDSCC. A total of 138 paired melanoma and non-tumor tissues and an additional

	58 melanoma tissues, including 101 AM tissues, were collected to construct the tissue micro-array (TMA).
Data exclusions	We excluded some patients with non maligant melanoma after verification by pathological experts.
Replication	All reported findings were replicated across multiple independent biological samples, including 12 AM samples in scRNA-seq, and 5 AM samples in ST-seq, including 12 AM samples in scRNA-seq and 5 AM samples in ST-seq.
Randomization	Patients with Acral melanoma were randomly recruited for this study. Patients with LN metastasis were assigned to the LN+AM group, while patients without LN metastasis were assigned to the LN-AM group.
Blinding	The researchers were blind to the clinical annotations at the point of biologic discovery.

Reporting for specific materials, systems and methods

We require information from authors about some types of materials, experimental systems and methods used in many studies. Here, indicate whether each material, system or method listed is relevant to your study. If you are not sure if a list item applies to your research, read the appropriate section before selecting a response.

Materials & experimental systems

n/a	Involved in the study
☐	☒ Antibodies
☐	☒ Eukaryotic cell lines
☒	☐ Palaeontology and archaeology
☐	☒ Animals and other organisms
☐	☒ Clinical data
☒	☐ Dual use research of concern
☒	☐ Plants

Methods

n/a	Involved in the study
☒	☐ ChIP-seq
☒	☐ Flow cytometry
☒	☐ MRI-based neuroimaging

Antibodies

Antibodies used

For IHC and multiplex IHC staining
Anti-MITF (1:2000, ABCAM, catalog number: ab303530, Clone number: EPR26363-10)
Anti-CD4 (1:300, Biolynx, catalog number: BX50023, Clone number: BP6028)
Anti-CD8 (1:300, Biolynx, catalog number: BX50036-C3, Clone number: BP6041)
Anti-NCAM1 (1:100, CST, catalog number: 3576S, Clone number: 123C3)
Anti-FGFBP2 (1:1000, SIGMA, catalog number: HPA039180)
Anti-FAP (1:50, ABCAM, catalog number: ab207178, Clone number: EPR20021)
Anti-CD31 (1:100, ABCAM, catalog number: ab28364)
Anti-Galectin-9 (1:300, NOVUS, catalog number: NBP2-45619, Clone number: OTI1D12)
anti-TIM3 (1:200, CST, catalog number: 45208S, Clone number: D5D5R)
anti-CD45 (1:200, Biolynx, catalog number: BX50068, Clone number: BP6073)
anti-CD44 (1:200, CST, catalog number: 3570S, Clone number: 156-3C11)
For WB analysis
Anti-MITF (1:1000, ABCAM, catalog number: ab303530, Clone number: EPR26363-10)
Anti-Histone-H3 (1:8000, Proteintech, catalog number: 17168-1-AP)

Validation

For IHC and multiplex IHC staining
Anti-MITF (1:2000, ABCAM, catalog number: ab303530, Clone number: EPR26363-10)
-https://www.abcam.cn/products/primary-antibodies/mitf-antibody-epr26363-10-ab303530.html
Anti-CD4 (1:300, Biolynx, catalog number: BX50023, Clone number: BP6028)
-https://www.biolynxtec.com/products/antibody/cd4.html
Anti-CD8 (1:300, Biolynx, catalog number: BX50036-C3, Clone number: BP6041)
-https://www.biolynxtec.com/products/antibody/cd8.html
Anti-NCAM1 (1:100, CST, catalog number: 3576S, Clone number: 123C3)
-https://www.cellsignal.com/products/primary-antibodies/ncam1-cd56-123c3-mouse-mab/3576?site-search-type=Products&N=4294956287&Ntt=3576s&fromPage=plp&_requestid=1524761
Anti-FGFBP2 (1:1000, SIGMA, catalog number: HPA039180)
-https://www.sigmaaldrich.cn/CN/zh/product/sigma/hpa039180
Anti-FAP (1:50, ABCAM, catalog number: ab207178, Clone number: EPR20021)
-https://www.abcam.cn/products/primary-antibodies/fibroblast-activation-protein-alpha-antibody-epr20021-ab207178.html
Anti-CD31 (1:100, ABCAM, catalog number: ab28364)
-https://www.abcam.cn/products/primary-antibodies/cd31-antibody-ab28364.html
Anti-Galectin-9 (1:300, NOVUS, catalog number: NBP2-45619, Clone number: OTI1D12)
-https://www.novusbio.com/products/galectin-9-antibody-oti1d12_nbp2-45619
anti-TIM3 (1:200, CST, catalog number: 45208S, Clone number: D5D5R)
-https://www.cellsignal.com/products/primary-antibodies/tim-3-d5d5r-xp-rabbit-mab/45208?site-search-type=Products&N=4294956287&Ntt=45208s&fromPage=plp&_requestid=357875
anti-CD45 (1:200, Biolynx, catalog number: BX50068, Clone number: BP6073)

-https://www.biolynxtec.com/products/antibody/cd45.html
anti-CD44 (1:200, CST, catalog number: 3570S, Clone number: 156-3C11)
-https://www.cellsignal.com/products/primary-antibodies/cd44-156-3c11-mouse-mab/3570?site-search-type=Products&N=4294956287&Ntt=3570s&fromPage=plp&_requestid=357955
For WB analysis
Anti-MITF (1:1000, ABCAM, catalog number: ab303530, Clone number: EPR26363-10)
-https://www.abcam.cn/products/primary-antibodies/mitf-antibody-epr26363-10-ab303530.html
Anti-Histone-H3 (1:8000, Proteintech, catalog number: 17168-1-AP)
-https://www.ptgcn.com/products/Histone-H3-Antibody-17168-1-AP.htm

Eukaryotic cell lines

Policy information about cell lines and Sex and Gender in Research

Cell line source(s)
B16F0 cell line was purchased from the cell bank of the Chinese Academy of Sciences (Shanghai, China).

Authentication
We did not performed the cell line authentication.

Mycoplasma contamination
The cell lines tested negative for mycoplasma contamination prior to cryopreservation.

Commonly misidentified lines (See ICLAC register)
None of the used cell lines are placed in the ICLAC register.

Animals and other research organisms

Policy information about studies involving animals; ARRIVE guidelines recommended for reporting animal research, and Sex and Gender in Research

Laboratory animals
Male C57BL/6 mice aged 5~6 weeks were purchased from the SLAC laboratory animal company (Shanghai, China). Mice were housed in a controlled environment with a 12-hour dark/light cycle and a temperature of 22°C. They had ad libitum access to food and water, which were autoclaved. Cages were changed weekly. The humidity was monitored and maintained between 30% and 70%.

Wild animals
No wild animals were used in the study.

Reporting on sex
All C57BL/6 mice are male.

Field-collected samples
No field collected samples were used in the study.

Ethics oversight
All experimental procedures were approved by the Animal Experimentation Ethics Committee of FDZSH (2023-252).

Note that full information on the approval of the study protocol must also be provided in the manuscript.

Clinical data

Policy information about clinical studies
All manuscripts should comply with the ICMJE guidelines for publication of clinical research and a completed CONSORT checklist must be included with all submissions.

Clinical trial registration
The bulk RNA-seq data of 26 primary AM samples were retrieved from GSE162682, and 101 AM patients' clinical data from FUZSH.

Study protocol
We applied the survival data of 26 AM patients to study levels of designated signatures in LN metastatic and non-metastatic AM patients, and to study the differences between designated signatures and prognosis of AM patients. The clinical data of melanoma patients from TMA of FUZSH were used to study the differences between designated signatures and prognosis of AM patients..

Data collection
The clinical data of 26 AM patients come from GSE162682 (www.ncbi.nlm.nih.gov/geo), and the clinical data of TMA comes from FUZSH.

Outcomes
We want to further elaborate the genomic mechanism behind clinical differences by connecting the results found in single cell data with the clinical information of patients.
